# PGC-1*α* buffers ROS-mediated removal of mitochondria during myogenesis

**DOI:** 10.1038/cddis.2014.458

**Published:** 2014-11-06

**Authors:** S Baldelli, K Aquilano, M R Ciriolo

**Affiliations:** 1 Scientific Institute for Research, Hospitalization and Health Care, Università Telematica San Raffaele Roma, Via di Val Cannuta 247, Rome, Italy; 2 Department of Biology, University of Rome “Tor Vergata”, Via della Ricerca Scientifica 1, Rome, Italy; 3 IRCCS San Raffaele, Via di Val Cannuta 247, Rome, Italy

## Abstract

Mitochondrial biogenesis and mitophagy are recognized as critical processes underlying mitochondrial homeostasis. However, the molecular pathway(s) coordinating the balance between these cellular programs is still poorly investigated. Here, we show an induction of the nuclear and mitochondrial peroxisome proliferator-activated receptor gamma, coactivator 1 alpha (PGC-1*α*) during myogenesis, which in turn co-activates the transcription of nuclear and mtDNA-encoded mitochondrial genes. We demonstrate that PGC-1*α* also buffers oxidative stress occurring during differentiation by promoting the expression of antioxidant enzymes. Indeed, by downregulating PGC-1*α,* we observed an impairment of antioxidants expression, which was accompanied by a significant reactive oxygen species (ROS) burst and increase of oxidative damage to proteins. In parallel, we detected a decrease of mitochondrial mass and function as well as increased mitophagy through the ROS/FOXO1 pathway. Upon PGC-1*α* downregulation, we found ROS-dependent nuclear translocation of FOXO1 and transcription of its downstream targets including mitophagic genes such as LC3 and PINK1. Such events were significantly reverted after treatment with the antioxidant Trolox, suggesting that PGC-1*α* assures mitochondrial integrity by indirectly buffering ROS. Finally, the lack of PGC-1*α* gave rise to a decrease in MYOG and a strong induction of atrophy-related ubiquitin ligases FBXO32 (FBXO32), indicative of a degenerative process. Overall, our results reveal that in myotubes, PGC-1*α* takes center place in mitochondrial homeostasis during differentiation because of its ability to avoid ROS-mediated removal of mitochondria.

Mitochondria are organelles that undergo an array of dynamic changes including biogenesis, selective degradation, fission, fusion and transport along the cell extremity.^1^ Fusion enables mitochondria to mix their metabolites, proteins and mitochondrial DNA (mtDNA).^2^ Fission is a mechanism that segregates components of mitochondria that are damaged. In addition, the conversion between fragmented and fused states allows mitochondria to rearrange and dispose damaged components trough mitophagy.^3^ In particular, when mitochondria are damaged PINK1 (PTEN-induced putative kinase 1) recruits PARK2 (parkin RBR E3 ubiquitin protein ligase) to depolarized mitochondria and promotes the PARK2-mediated ubiquitination of outer mitochondrial membrane proteins. Next, the sequestosome SQSTM1 binds ubiquitinated proteins and targets them to LC3I-II (microtubule-associated protein 1 light chain 3 alpha) to promote selective degradation.^3^ An impairment of these processes has been implicated in aging, neurodegeneration and muscular atrophy.^4^

Skeletal muscle represents an interesting environment, as the plasticity of mitochondria is a key factor in cellular metabolic adaptation to exercise or inactivity.^5^ Both cardiac and skeletal muscle have a limited proliferative capacity, thus the regulation of their size and functionality is based on protein and organelle turnover. Notwithstanding, the mechanism by which mitochondrial dynamic is regulated in skeletal muscle remains to be understood.

The transcriptional coactivator peroxisome proliferator-activated receptor *γ* coactivator-1 *α* (PGC-1*α*) is considered as the ‘master regulator' of mitochondrial biogenesis, which improves the expression of nuclear genes encoding mitochondrial proteins.^6^ We have demonstrated that PGC-1*α*, which has been considered a nuclear protein, is also distributed inside mitochondria where it forms a multiprotein complex with transcription factor A mitochondrial (TFAM) on mtDNA.^6, 10, 11^ PGC-1*α* is required for the induction of many antioxidant-detoxifying enzymes.^12, 13^ PGC-1*α* is also a modulator of multiple pathways coordinating skeletal muscle tissue adaptation to exercise.^14, 15, 16^ Transgenic mice that express PGC-1*α* in fast glycolytic muscle show a switch towards oxidative metabolism and increased mitochondrial content.^17^ Moreover, PGC-1*α* has been implicated in the regulation of skeletal muscle mass, particularly in condition of muscle atrophy.^18, 19^ Elevated PGC-1*α* and PGC-1*β* levels prevent the induction of autophagy and atrophy-specific ubiquitin ligase by a constitutively active forkhead transcription factor 3 (FOXO3).^19, 20^ Recent studies have demonstrated that FOXO1 and FOXO3a regulate autophagy in a reactive oxygen species (ROS)-dependent manner.^21, 22^ These findings indicate that PGC-1*α* has a role in regulating autophagy as well as mitochondrial biogenesis. It remains unclear how this coactivator switches on/off the induction of autophagy to maintain muscle mass by means of transcriptional control.

In this work, we demonstrate that PGC-1*α* is a fundamental regulator of mitochondrial turnover in differentiating myoblasts, inhibiting mitophagy in favor of mitochondrial biogenesis. We also discovered that PGC-1*α*-mediated inhibition of mitophagy is elicited by its capacity to counteract oxidative stress and maintain the expression of antioxidants, thus suppressing the signaling axis consisting in the ROS/FOXO1-mediated activation of mitophagic genes.

## Results

### PGC-1*α* induction sustains mitochondrial homeostasis during myogenesis

**Figure 1:**
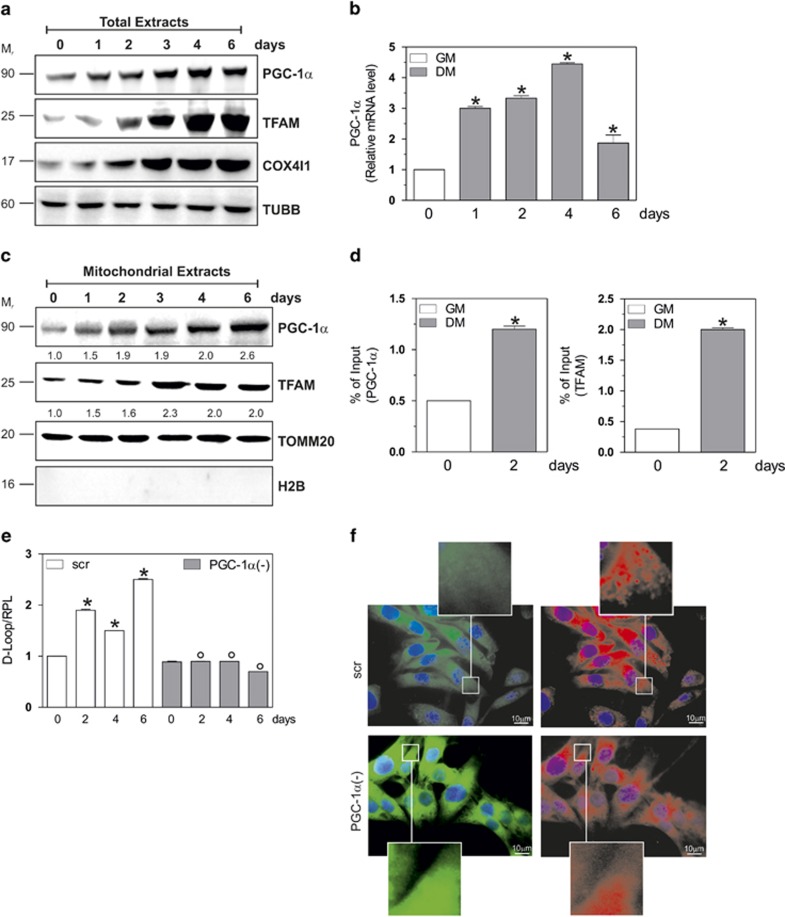
Nuclear and mitochondrial PGC-1*α* induction is necessary to mitochondrial functionality during myogenesis. C2C12 cells were differentiated in DM for the indicated days. (**a**) Twenty micrograms of total proteins were loaded for western blot analysis of PGC-1*α*, TFAM and COX4I1. TUBB was used as the loading control. (**b**) Total RNA was isolated, and relative mRNA level of PGC-1*α* was analyzed by RT-qPCR. Data are expressed as means±S.D. (*n*=6, *P<0.001). (**c**) Twenty micrograms of mitochondrial proteins extracts were loaded for western blot analysis of PGC-1*α* and TFAM. TOMM20 was used as the loading control. To exclude the presence of nuclear contaminants, the nitrocellulose was probed with H2B antibody. Numbers indicate the density of immunoreactive bands calculated using the Software Quantity one (Bio-Rad) and reported as the ratio of PGC-1*α*/TOMM20 and TFAM/TOMM20. (**d**) mtDNA-IP assay was carried out on cross-linked mitochondria using PGC-1*α* (*left panel*) or TFAM (*right panel*) antibody followed by qPCR analysis of D-Loop TFAM *consensus* sequence. Data are expressed as means±S.D. (*n*=4, *P<0.001). (**e**) C2C12 cells were transfected with scramble (scr) or PGC-1*α* siRNA (PGC-1*α*(−)), differentiated in DM for the indicated days. DNA was extracted and relative mtDNA content was assayed by analyzing D-Loop level through qPCR. D-Loop value was normalized to ribosomal protein large subunit (RPL). Data are expressed as means±S.D. (*n*=5, *P<0.001 *versus* day 0 scr cells; °*P*<0.001 *versus* scr cells). (**f**) Cells were grown on glass coverslips, differentiated until day 2 and incubated for 30 min with 1 *μ*g/ml JC-1. Paraformaldehyde-fixed cells were stained with Hoechst 33342 and subjected to fluorescence microscopy. Magnitude insets are shown at the bottom and top of each picture. Images reported are from one experiment representative of three that gave similar results. Immunoblots reported in the figures are representative of at least four experiments that gave similar results

In this work, we aimed at characterizing the role of PGC-1*α* in mitochondrial dynamic during C2C12 myogenesis. Figures 1a and b and Supplementary Figure 1a show that PGC-1*α* protein and mRNA increased over time during myogenesis and this event is accompanied by the induction of the expression of its nuclear target genes (i.e., TFAM and COX4I1). Subsequently, we investigated whether the mitochondrial content of PGC-1*α* could be modulated during myogenesis. As shown in Figure 1c, a progressive increase of PGC-1*α* was observed in mitochondria purified at different stages of differentiations. This effect was accompanied by an increase of TFAM in mitochondria (Figure 1c). We determined an improved association of PGC-1*α* and TFAM with the D-Loop region of mtDNA as assessed by mt-ChIP assay (Figure 1d). This event resulted in an enhanced expression of TFAM-encoded genes, such as MT-CO1 and MT-ATP6 (Supplementary Figure 1b).

To confirm the role of PGC-1*α* in coordinating mitochondrial turnover, we downregulated its expression by means of siRNA transfection. To this end, C2C12 cells were transfected with a siRNA against PGC-1*α* (PGC-1*α*(−)) or with a scramble siRNA (scr). Downregulation of PGC-1*α* was successfully achieved as both its mRNA and protein content were significantly decreased (Supplementary Figure 2a). We then analyzed the mRNA levels of MT-CO1 and MT-ATP6 and we found that PGC-1*α* downregulation significantly blocked their expression (Supplementary Figure 2b). Supplementary Figure 2b also shows that other PGC-1*α* downstream markers of mitochondrial biogenesis (such as NRF1, NRF2, POLG, POLRMT) remained at basal level in PGC-1*α*(−) cells. This event was also confirmed by evaluating the protein level of MT-CO1 and TFAM (Supplementary Figure 2c). Coherently, the proliferation of mitochondria was restrained, as evaluated by analyzing mtDNA content through qPCR (Figure 1e).

To unravel whether PGC-1*α* was involved in the maintenance of mitochondrial activity, we analyzed mitochondrial membrane potential (ΔΨ_m_) by the fluorochrome JC-1. JC-1 is a lipophilic cationic dye that incorporates into mitochondria, where it forms monomers (fluorescence in green, 527 nm) detecting depolarized mitochondria, or J-aggregates (fluorescence in red, 590 nm) evidencing polarized mitochondria. At day 2 of differentiation, we found that PGC-1*α* deficiency reduced J-aggregates and increased its monomers, suggesting a raise of depolarized mitochondria (Figure 1f).

### PGC-1*α* downregulation induces mitophagy during myogenesis

**Figure 2:**
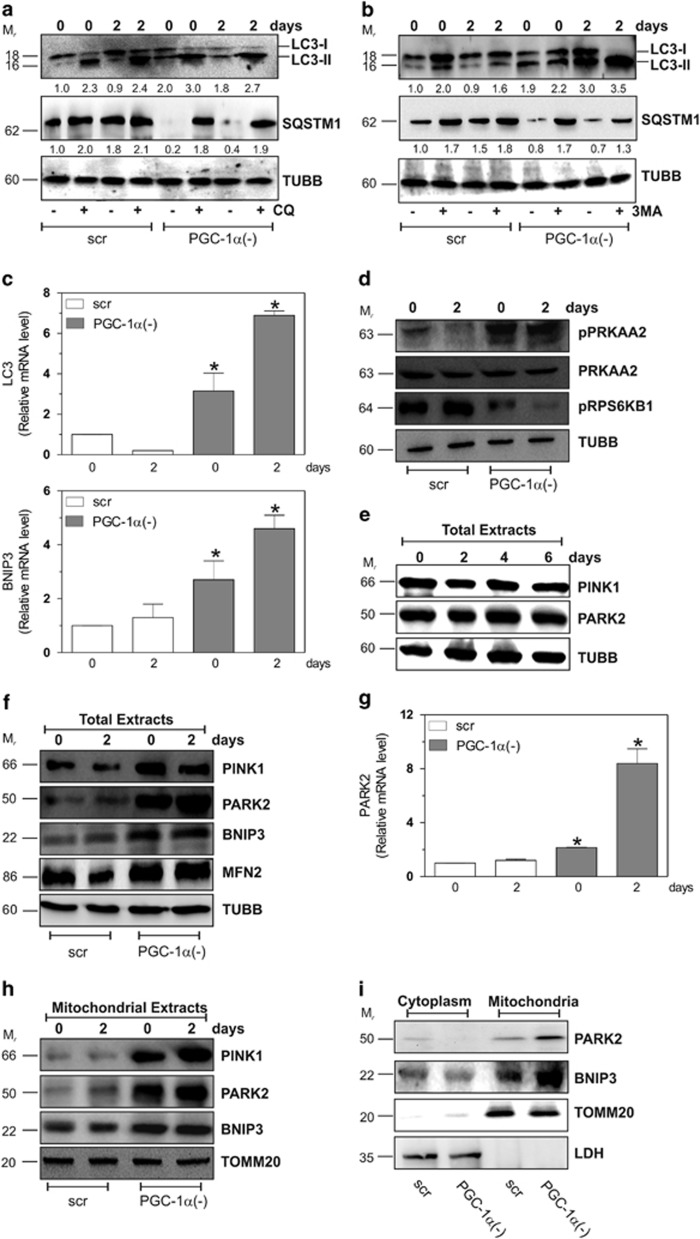
PGC-1*α* buffers mitophagy during myogenesis. C2C12 cells were transfected with scramble (scr) or PGC-1*α* siRNA (PGC-1*α*(−)), differentiated in DM for the indicated days. (**a**) C2C12 cells were treated with 5 *μ*M CQ and 20 *μ*g of total proteins extracts were loaded for western blot analysis of LC3I-II and SQSTM1. TUBB was used as the loading control. Numbers indicate the density of immunoreactive bands calculated using the Software Quantity one (Bio-Rad) and reported as the ratio of LC3II/TUBB and SQSTM1/TUBB. (**b**) C2C12 cells were treated with 5 mM 3MA and 20 *μ*g of total proteins extracts were loaded for western blot analysis of LC3I-II and SQSTM1. TUBB was used as the loading control. Numbers indicate the density of immunoreactive bands calculated using the Software Quantity one (Bio-Rad) and reported as the ratio of LC3II/TUBB and SQSTM1/TUBB. (**c**) Total RNA was isolated, and relative mRNA level of LC3 and BNIP3 were analyzed by RT-qPCR. Data are expressed as means±S.D. (*n*=6, *P<0.001 *versus* day 0 scr cells). (**d**) Twenty micrograms of total proteins extracts were loaded for western blot analysis of pPRKAA2, PRKAA2 and pRPS6KB1. TUBB was used as the loading control. (**e**) Twenty micrograms of total proteins were loaded for western blot analysis of PINK1 and PARK2. TUBB was used as the loading control. (**f**) Twenty micrograms of total proteins were loaded for western blot analysis of PINK1, PARK2, BNIP3 and MFN2. TUBB was used as the loading control. (**g**) Total RNA was isolated, and relative mRNA level of PARK2 were analyzed by RT-qPCR. Data are expressed as means±S.D. (*n*=4, *P<0.001 *versus* day 0 scr cells). (**h**) Twenty micrograms of mitochondrial proteins extracts were loaded for western blot analysis of PINK1, PARK2 and BNIP3. TOMM20 was used as the loading control. (**i**) Twenty micrograms of mitochondrial and cytoplasmatic proteins extracts were loaded for western blot analysis of PARK2 and BNIP3. TOMM20 and LDH were used as the loading control. Immunoblots reported in the figures are representative of at least four experiments that gave similar results

The turnover of mitochondria is assured by a complex array of processes such as proliferation and selective elimination by mitophagy.^23^ However, there are no strong evidence about the molecular mechanism(s) that coordinate the balance between mitochondrial biogenesis and mitophagy. Thus, we examined whether the mitochondrial-shaping machinery was altered by the downregulation of PGC-1*α*. We first analyzed the level of autophagy during differentiation. Supplementary Figure 2d shows that no significant alterations in some autophagic hallmarks such as BNIP3, LC3I-II and SQSTM1 were detected. On the contrary, after PGC-1*α* downregulation, we observed a significant modulation of these proteins. Indeed, Figures 2a and b demonstrate a significant rise in the lipidated form (LC3II) of LC3I-II and a concomitant decrease of SQSTM1 protein level in PGC-1*α*(−) cells, implying an higher rate of autophagic flux. This assumption was confirmed by treatment with 5 *μ*M chloroquine (CQ) and 5 mM 3-methyladenine (3MA). As shown in Figures 2a and b, either CQ or 3MA treatment led to an higher accumulation of LC3II in PGC-1*α*(−) with respect to scr cells. Similarly, either CQ or 3MA treatments induced an increase of SQSTM1 protein level after PGC-1*α* downregulation (Figures 2a and b). To further demonstrate an enhanced autophagy during myogenesis in the absence of PGC-1*α*, we evaluated the mRNA levels of LC3 and BNIP3. As reported in Figure 2c, although LC3 and BNIP3 did not undergo any changes in scr cells, their mRNA were significantly increased upon PGC-1*α* downregulation. The more efficient induction of autophagy in PGC-1*α*(−) cells was also substantiated by the dramatic upregulation of the phosphorylated form of PRKAA2, whereas its basal level remained unchanged (Figure 2d). Indeed, PRKAA2 has been previously suggested to activate autophagy under energetic stress.^24, 25, 26^ To define a possible link between PRKAA2 and MTOR under our experimental conditions, we used an anti-phospho RPS6KB1 antibody, which is a direct substrate of MTOR. As shown in Figure 2d, the phosphorylation of RPS6KB1 was clearly observed in scr cells, whereas it significantly decreased after downregulation of PGC-1*α*.

Given that PGC-1*α* downregulation led to a significant drop of ΔΨ, we moved at investigating whether PGC-1*α* can also induce an efficient clearance of damaged mitochondria through mitophagy. We looked at some mitophagic markers (i.e., PINK1 and PARK2) and we found that they remained unaltered during myogenesis (Figure 2e). Conversely, PGC-1*α* deficiency resulted in a raise of PINK1 and PARK2 indicating the induction of mitophagy (Figures 2f and g). Moreover, we found an increase of mitofusin 2 (MFN2), a marker of mitochondrial fusion, a dynamic process also occurring in order to buffer mitochondrial damages (Figure 2f). Selective degradation of dysfunctional mitochondria occurs through the stabilization of PINK1 on the outer membrane, with the consequent recruitment of PARK2. We did not detect any changes in PINK1 and PARK2 protein levels in mitochondria of scr cells during differentiation (Figure 2h). Contrarily, in mitochondria of PGC-1*α*(−) cells, a large increase in these proteins was detected (Figure 2h). Also, mitochondrial content of BNIP3 was significantly augmented in these cells (Figure 2h). Finally, the activation of mitophagy has also been demonstrated comparing mitochondrial and cytoplasmatic fraction. In fact, Figure 2i shows a cytoplasmatic decrease of PARK2 and BNIP3 protein levels, which was paralleled by an increase in mitochondrial fraction.

**Figure 3:**
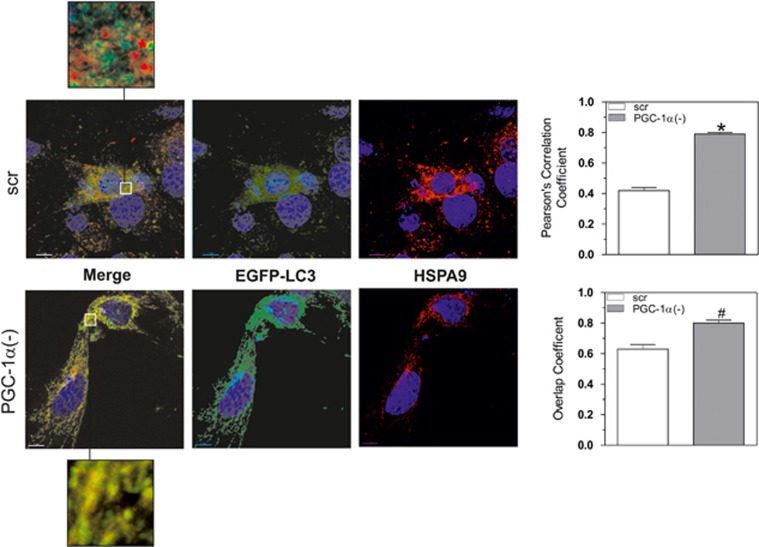
PGC-1*α* deficiency induces mitophagy in C2C12 cells. C2C12 cells were co-transfected with scramble (scr) or PGC-1*α* siRNA (PGC-1*α*(−)) and with EGFP-LC3. At day 0, C2C12 cells were fixed with paraformaldehyde and immunostained with mouse anti-HSPA9, Hoechst 33342 and then visualized by confocal microscopy. Scale bar 7 *μ*m. Overlap and Pearson's coefficients were calculated by JACoP (plugin of ImageJ Software) in at least 10 different images. Data are expressed as means±S.D. (*n*=4, *P<0.001, ^#^*P*<0.01 *versus* scr cells). Yellow in the merged image indicates co-localization of EGFP-LC3 with mitochondria (HSPA9). Magnitude insets are shown at the bottom and top of each picture. Images reported are from one experiment representative of three that gave similar results

Subsequently, C2C12 cells were co-transfected with siRNA against PGC-1*α* and with a vector expressing the LC3 conjugated with EGFP (EGFP-LC3). By confocal microscopy, we then monitored the presence of dotted green fluorescence that is typical of active autophagosome formation. Figure 3 highlights that a greater number of green spots were present in PGC-1*α*(−) cells with respect to scr cells. Co-localization analysis of EGFP-LC3 and mitochondria stained with HSPA9 showed that autophagosomes more efficiently co-localized with mitochondria in PGC-1*α*(−) cells, confirming the pivotal role of PGC-1*α* in maintaining mitochondrial mass.

### PGC-1*α* counteracts the ROS-mediated mitophagy

**Figure 4:**
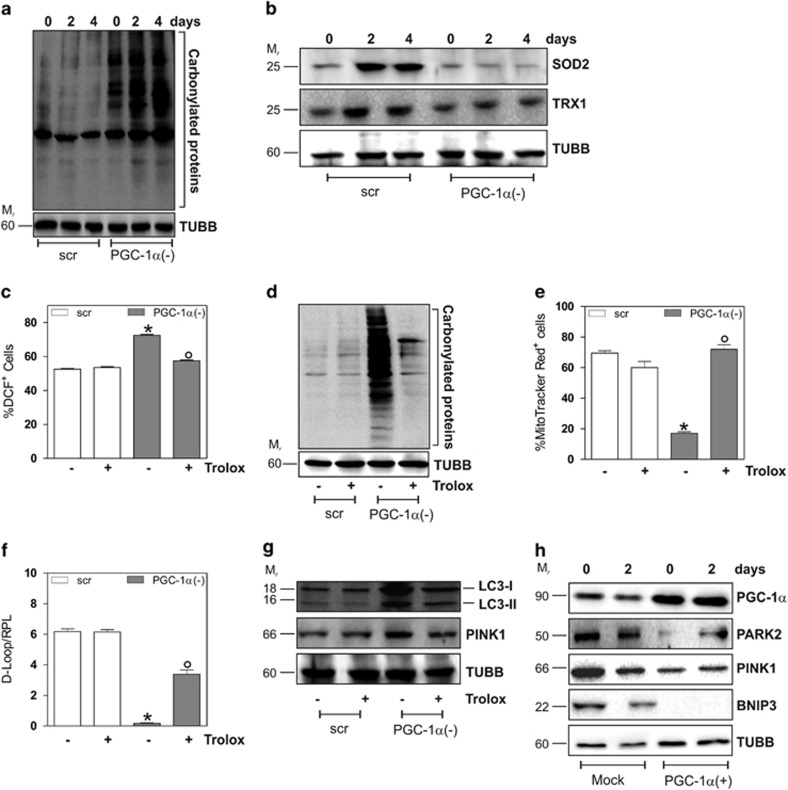
PGC-1*α* downregulation is associated with an increase of oxidative stress and alterations of mitochondrial homeostasis. C2C12 cells were transfected with scramble (scr) or PGC-1*α* siRNA (PGC-1*α*(−)) and differentiated in DM for the indicated days. (**a**) Twenty micrograms of total proteins were derivatized with DNP and carbonylation was detected by western blot with DNP antibody. TUBB was used as the loading control. (**b**) Twenty micrograms of total proteins were loaded for western blot analysis of SOD2 and TRX1. TUBB was used as the loading control. (**c**) C2C12 cells were treated with 200 *μ*M Trolox and maintained throughout the experiment. ROS production was assayed by cytofluorimetric analysis after DCF-DA staining. ROS level was reported as the percentage of DCF-positive cells and expressed as means ±S.D. (*n*=3; *P<0.001 *versus* scr cells; °*P*<0.01 *versus* PGC-1*α*(−) cells). (**d**) Twenty micrograms of total proteins were derivatized with DNP and carbonylation was detected by western blot with DNP antibody. TUBB was used as the loading control. (**e**) C2C12 cells were incubated with MitoTrackerRed for 30 min and mitochondrial content was assayed by cytofluorimetric analysis. Data are expressed as percentage of MitoTrackerRed-positive cells (*n*=4; *P<0.001 *versus* scr cells; °*P*<0.01 *versus* PGC-1*α*(−) cells). (**f**) DNA was extracted and relative mtDNA content was assayed by analyzing the D-Loop level through qPCR. D-Loop value was normalized to RPL. Data are expressed as means±S.D. (*n*=5, *P<0.001 *versus* scr cells; °*P*<0.001 *versus* PGC-1*α*(−) cells). (**g**) Twenty micrograms of total proteins were loaded for western blot analysis of LC3I-II and PINK1. TUBB was used as the loading control. Immunoblots reported in the figures are representative of at least four experiments that gave similar results. (**h**) C2C12 cells were transfected with empty vector (Mock) or pSV-PGC-1*α* vector (PGC-1*α*(+)) and differentiated in DM for the indicated days. Twenty micrograms of total proteins were loaded for western blot analysis of PGC-1*α*, PARK2, PINK1 and BNIP3. TUBB was used as the loading control

Previous reports show the correlation between production of ROS and the induction of mitophagic/autophagic processes.^27^ It has also been demonstrated that PGC-1*α* is a broad and powerful regulator of ROS metabolism by preventing oxidative stress.^12, 13, 28^ Therefore, we speculated that an increased ROS production might be involved in the exacerbation of mitophagy in the absence of PGC-1*α* during differentiation. To this end, we measured the extent of protein oxidation as hallmark of increased ROS production. As shown in Figure 4a, a time-dependent increase in total protein carbonyls was observed in PGC-1*α*(−) cells with respect to scr cells. Moreover, we analyzed the protein levels of SOD2 and TRX1, which are notably induced by PGC-1*α*.^13, 28^ The western blot and densitometric analysis reported in Figure 4b and Supplementary Figure 2e show that SOD2 and TRX1 increased during myogenesis. Expectedly, a decrement of these antioxidants was observed after downregulation of PGC-1*α*. Coherently, by monitoring the production of ROS through cytofluorimetric analysis, we found an enhanced ROS flux in PGC-1*α*(−) cells (Figure 4c), which was abolished by treatment with the ROS-scavenger Trolox (200 *μ*M). Figure 4c shows that Trolox was able to significantly restore the physiological ROS concentration in PGC-1*α*(−) cells, suggesting that the increase of ROS levels was elicited by the downregulation of PGC-1*α*. As consequence of ROS buffering, Trolox also dampened the increase of protein carbonyls (Figure 4d). We next examined the effect of Trolox on ΔΨ after staining with the fluorescent probe MitoTrackerRed, which selectively accumulates in polarized mitochondria. In line with the results obtained with JC-1, PGC-1*α* downregulation induced a decrease of ΔΨ at day 2 of differentiation with respect to scr cells (Figure 4e). Notably, Trolox allowed a complete recovery of ΔΨ in PGC-1*α*(−) cells, suggesting that in the absence of PGC-1*α* the increased ROS production is responsible for mitochondrial dysfunction. On the contrary, Trolox only partially recovered the decrease of mtDNA upon PGC-1*α* deficiency, suggesting that PGC-1*α* directly acts as a modulator of mtDNA copy number (Figure 4f). By preventing ROS-mediated mitochondrial damage, Trolox also reduced the induction of mitophagy in PGC-1*α*(−) to level comparable to that of scr cells, as demonstrated by decreased LC3II and PINK1 protein levels (Figure 4g). To finally prove that PGC-1*α* is a powerful regulator of mitophagy during myogenesis, we transfected C2C12 cells with PGC-1*α* cDNA. Figure 4h shows that PGC-1*α* overexpression was able to decrease the protein level of Parkin, PINK1 and BNIP3 at day 0 and 2 of differentiation.

### FOXO1 promotes mitophagy through the transcription of PINK1 and LC3 genes in a ROS-dependent manner

**Figure 5:**
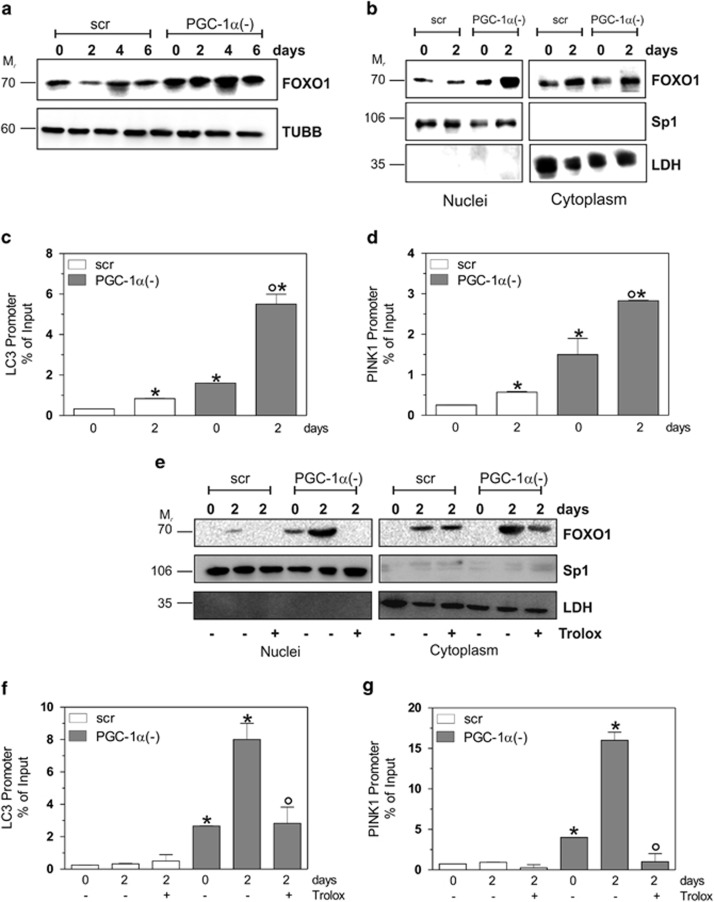
FOXO1 promotes mitophagy in a ROS-dependent manner during myogenesis. C2C12 cells were transfected with scramble (scr) or PGC-1*α* siRNA (PGC-1*α*(−)) and differentiated in DM for the indicated days. (**a**) Twenty micrograms of total proteins were loaded for western blot analysis of FOXO1. TUBB was used as the loading control. (**b**) Twenty micrograms of nuclear and cytoplasmatic proteins were loaded for western blot analysis of FOXO1. Sp1 and LDH were used as markers of fraction purity and as the loading control. (**c**, **d**) At day 2 of myogenesis, ChIP assay was carried out on cross-linked nuclei using LC3II (*upper panel*) or PINK1 (*bottom panel*) antibody followed by qPCR analysis of FOXO1 *consensus* sequence. Data are expressed as means±S.D. (*n*=4, *P<0.001 *versus* scr cells; °*P*<0.001 *versus* day 0 PGC-1*α*(−) cells). (**e**) C2C12 cells were treated with 200 *μ*M Trolox and maintained throughout the experiment (2 days). Twenty micrograms of nuclear and cytoplasmatic proteins were loaded for western blot analysis of FOXO1. Sp1 and LDH were used as markers of fraction purity and as the loading control. (**f**, **g**) C2C12 cells were treated with 200 *μ*M Trolox and maintained throughout the experiment (2 days). ChIP assay was carried out on cross-linked nuclei using LC3II (*upper panel*) or PINK1 (*bottom panel*) antibody followed by qPCR analysis of FOXO1 *consensus* sequence. Data are expressed as means±S.D. (*n*=6, *P<0.001 *versus* scr cells; °*P*<0.001 *versus* untreated day 2 PGC-1*α*(−) cells). Immunoblots reported in the figures are representative of at least four experiments that gave similar results

To investigate the signaling pathway(s) involved in PGC-1*α*-mediated buffering of mitophagy, we focused on the FOXO1 transcription factor, which can regulate the expression of autophagy/mitophagy-related genes in response to an increase of ROS.^27, 29^ Hence, we examined FOXO1 protein level during differentiation after PGC-1*α* downregulation. A strong increase of FOXO1 was detected in total extracts of PGC-1*α*(−) cells with respect to scr cells (Figure 5a). Subsequently, we investigated the possible shuttling of FOXO1 between cytoplasm and nuclei. As reported in Figure 5b, a significant amount of FOXO1 protein accumulated in the nuclei of PGC-1*α*(−) cells already at day 0, and differentiation further enhanced its nuclear recruitment. We asked whether FOXO1 was directly involved in the transcriptional regulation of mitophagy/autophagy-related genes^30, 31, 32^ and we firstly focused on LC3 and PINK1. In particular, we performed a ChIP assay to quantify the binding of FOXO1 to LC3 and PINK1 promoters. qPCR analysis revealed that FOXO1 occupancy on LC3 and PINK1 promoters was markedly increased in PGC-1*α*(−) cells (Figures 5c and d). To assess whether ROS accumulation could be involved in the modulation of FOXO1 activation and consecutively in the mitophagy induction, we analyzed the effects of Trolox on FOXO1 subcellular redistribution. Figure 5e shows that Trolox significantly lowered FOXO1 nuclear migration both in scr and PGC-1*α*(−) cells. As a result, LC3 and PINK1 promoters were not engaged by FOXO1 upon Trolox treatment in PGC-1*α*(−) cells (Figures 5f and g), suggesting that PGC-1*α* by maintaining ROS balance, is able to indirectly inhibit FOXO1-mediated mitophagy.

**Figure 6:**
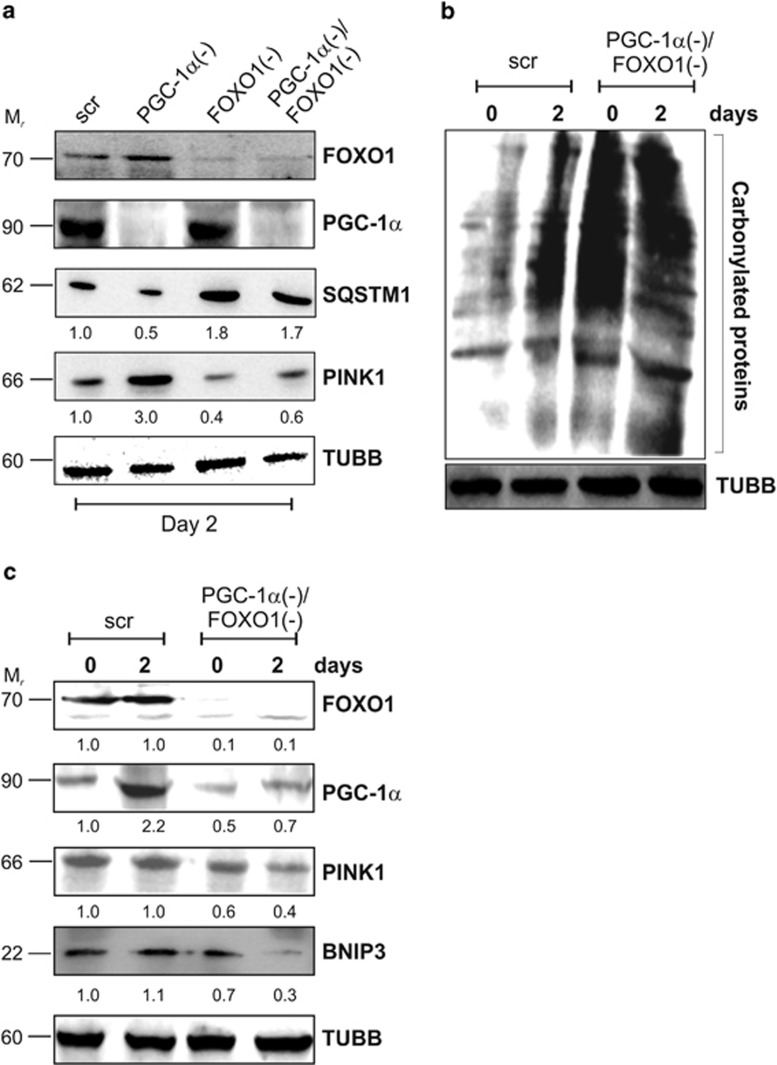
Downregulation of FOXO1 enables mitophagy induction. C2C12 cells were transfected with scramble (scr), PGC-1*α* and/or FOXO1 siRNA (PGC-1*α*(−), FOXO1(−) and PGC-1*α*/FOXO1(−)) and differentiated in DM for the indicated days. (**a**) Twenty micrograms of total proteins were loaded for western blot analysis of FOXO1, PGC-1*α*, SQSTM1 and PINK1. TUBB was used as the loading control. Numbers indicate the density of immunoreactive bands calculated using the Software Quantity one (Bio-Rad) and reported as the ratio of SQSTM1/TUBB and PINK1/TUBB. (**b**) Twenty micrograms of total proteins were derivatized with DNP and carbonylation was detected by western blot with DNP antibody. TUBB was used as the loading control. (**c**) Twenty micrograms of total proteins were loaded for western blot analysis of FOXO1, PGC-1*α*, PINK1 and BNIP3. TUBB was used as the loading control. Numbers indicate the density of immunoreactive bands calculated using the Software Quantity one (Bio-Rad) and reported as the ratio of proteins/TUBB. Immunoblots reported in the figures are representative of at least four experiments that gave similar results

To confirm the role of PGC-1*α* and FOXO1 in modulating mitophagy, we co-transfected cells with siRNA against PGC-1*α* and FOXO1. As reported in Figure 6a, FOXO1 downregulation inhibited the expression of the mitophagic gene PINK1 and led to an accumulation of SQSTM1, suggesting that basal autophagy was impaired. The mitophagy process was efficiently abrogated when FOXO1 was downregulated in PGC-1*α*(−) cells. In particular, PGC-1*α*(−)/FOXO1(−) cells displayed a significant decrease of PINK1 and a concomitant increase of SQSTM1 protein levels with respect to scr and PGC-1*α*(−) cells. As reported in Figure 6b, an increase of carbonylated proteins was still observed in PGC-1*α*(−)/FOXO1(−) cells. However, even though ROS were increased in cells downregulating both FOXO1 and PGC-1*α*, the mitophagy process was efficiently dampened. Indeed, a reduction of BNIP3 protein levels was observed (Figure 6c) in PGC-1*α*(−)/FOXO1(−) cells, confirming the positive role of FOXO1 in autophagy/mitophagy induction.

### PGC-1*α* inhibition and FOXO1 activation cause degeneration of C2C12 myotubes

**Figure 7:**
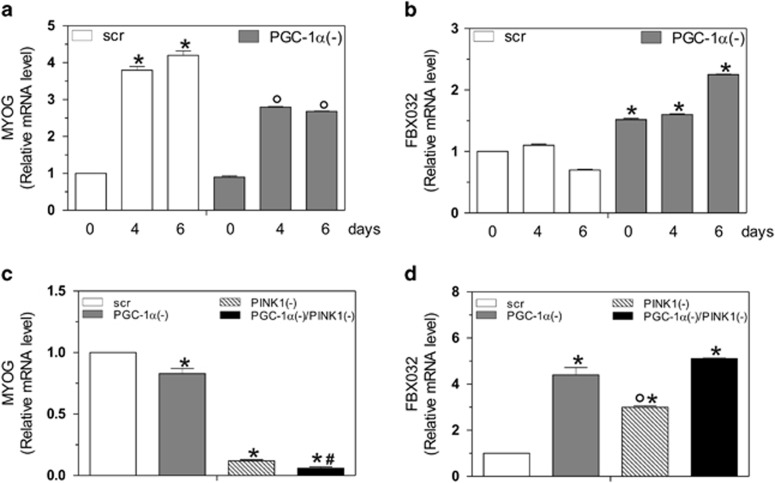
Depletion of PGC-1*α* inhibits myogenin and induces FBXO32 expression. C2C12 cells were transfected with scramble (scr) or PGC-1*α* siRNA (PGC-1*α*(−)) and differentiated in DM for the indicated days. (**a**, **b**) Total RNA was isolated, and relative mRNA levels of MYOG (**a**) and FBXO32 (**b**) were analyzed by RT-qPCR. Data are expressed as means±S.D. (*n*=4, *P<0.001 *versus* day 0 scr cells; °*P*<0.001 *versus* scr cells). (**c**, **d**) C2C12 cells were transfected with scramble (scr), PGC-1*α* and/or PINK1 siRNA (PGC-1*α*(−), PINK1(−) and PGC-1*α*/PINK1(−)) and differentiated for 2 days. Total RNA was isolated, and relative mRNA levels of MYOG (**c**) and FBXO32 (**d**) were analyzed by RT-qPCR. Data are expressed as means±S.D. (*n*=4, *P<0.001 *versus* scr cells; °*P*<0.001 *versus* PGC-1*α*(−) cells, PGC-1*α*(−)/PINK1(−) cells and ^#^*P*<0.001 *versus* PINK1(−) cells)

Given the importance of mitochondrial homeostasis during myogenesis, we hypothesized that PGC-1*α* deficiency could influence the integrity of myotubes. Thus, to identify the critical role of PGC-1*α* in differentiation and prevention of myotube degeneration, we analyzed the mRNA level of MYOG, a muscle-specific basic-helix-loop-helix transcription factor.^33^ As shown in Figure 7a, the expression of MYOG increased during myogenesis in scr cells. Notably, downregulation of PGC-1*α* attenuated the increase of MYOG levels, indicating that the differentiation program was retarded in PGC-1*α*(−) cells. To analyze the physiological state of myotubes in the absence of PGC-1*α*, we looked at the mRNA content of FBXO32, an ubiquitin ligase involved in atrophy. ^34^ After 6 days of differentiation, PGC-1*α*(−) cells showed a significant increase of FBXO32 mRNA with respect to scr cells (Figure 7b), suggesting that the ablation of PGC-1*α* and the consequent activation of FOXO1 commits C2C12 myotubes to a degenerative program.

Finally, to investigate whether the activation of mitophagy is a detrimental mechanism in our experimental conditions, we co-transfected cells with siRNA against PGC-1*α* and PINK1. We then analyzed the mRNA levels of MYOG and FBX032 by RT-qPCR analysis. PGC-1*α* deficiency induced alteration of the differentiation program and an induction of muscle degeneration that was exacerbated by the co-transfection with siPINK1 (Figures 7c and d). Moreover, the sole inhibition of PINK1 resulted in muscle degeneration even if at a lower extent (Figures 7c and d). These results indicate that during differentiation, basal mitophagy is essential to maintain mitochondrial homeostasis in order to avoid autophagic cell death.

## Discussion

The importance of PGC-1*α* in orchestrating mitochondrial biogenesis is to date well established. ^6, 7, 11, 35^ However, its role in the context of mitophagy and mitochondrial dynamics is still a debated matter of research. Recently, it has been demonstrated that the induction of autophagy does not necessarily affect mitochondrial content. Indeed, autophagy can be accompanied by an increase of PGC-1*α*, which maintains an adequate mitochondrial network.^36^ However, the molecular mechanism(s) that characterizes this phenomenon has not been fully delineated. Moreover, a dynamic co-regulation of PGC-1*α* with DCN (decorin), a tumor suppressor gene evoking mitophagy, has been demonstrated in breast carcinoma.^37^ Also, in the same work, the signaling pathway and the specific role of PGC-1*α* in governing mitophagy remains elusive and limited to cancer cells. To the best of our knowledge, here, we have revealed the molecular players linking mitophagy and biogenesis, identifying PGC-1*α* as a ‘guardian' of mitochondrial homeostasis, which maintains the integrity of mitochondrial functionality and the normal mitophagy flux during myogenesis.

Skeletal muscle is a tissue with high energy demand, in which mitochondrial plasticity and mitophagy have a fundamental role in the homeostasis and differentiation of these cells. The rate of mitophagic process in skeletal muscle must be well balanced in order to ensure a correct differentiation.^38^ Our data show that PGC-1*α* guarantees the production of ‘healthy' mitochondria and a basal flow of mitophagy during myogenesis. Through this mechanism, PGC-1*α* assures a correct equilibrium between mitophagy and mitochondrial biogenesis blocking an excessive ROS production, which could lead to an extreme activation of mitophagy flux, and muscle mass degeneration. In fact, our analyses of mitophagy markers showed that they were not modulated during normal differentiation and that only PGC-1*α* deficiency was associated with increased mitochondrial fusion and FOXO1-mediated mitophagy.

We found that PGC-1*α* capacity to shift the balance toward mitochondrial biogenesis relies upon its antioxidant function. PGC-1*α* is a known regulator of ROS scavenging enzymes,^12, 13^ providing a potential pathway for the manipulation of mitophagy. In fact, many studies demonstrate that the autophagy can be induced in response to intracellular ROS.^27, 39, 40^ Our results show that the downregulation of PGC-1*α* causes an increase of intracellular ROS levels and carbonylated proteins and a decrease of antioxidant enzymes. Accordingly, the use of the antioxidant Trolox in PGC-1*α* deficient cells avoids the accumulation of ROS and the drop of ΔΨ, confirming the role of ROS in mediating mitochondrial damage and induction of mitophagy. In line with these findings, the overexpression of PGC-1*α* results in a decrement of mitophagic markers, proving that this coactivator is necessarily involved in the prevention of excessive mitophagic rate during myogenesis.

It has been demonstrated that FOXO1 is able to modulate the transcription of genes involved in autophagic/mitophagic processes.^21, 22, 30, 31, 32, 41, 42, 43^ In line with this evidence, we found that FOXO1 can activate mitophagy as consequence of PGC-1*α* deficiency. Indeed, PGC-1*α* downregulation was associated with FOXO1 accumulation into nuclei and enhanced binding to PINK1 and LC3 promoters with consequent increase of their mRNA expression. These findings were coincident with the decrement of phosphorylated RPS6KB1 and an increase of phosphorylated PRKAA2 in PGC-1*α* lacking cells, suggesting that the signaling axis that governs the mitophagic process proceeds *via* the PRKAA2/MTOR pathway. The induction of mitophagy, in the absence of PGC-1*α*, was confirmed by the formation of mitophagosomes as evidenced by the co-localization of EGFP-LC3 puncta with mitochondrial HSPA9 fluorescence. The involvement of FOXO1 on mitophagy upon PGC-1*α* deficiency was finely demonstrated by experiments where a concomitant downregulation of FOXO1 and PGC-1*α* was carried out. Despite the increase of carbonylated proteins, the downregulation of FOXO1 and PGC-1*α* effectively abrogated the mitophagy induction. Our data strongly suggest that FOXO1 activation is necessarily required to increase the transcription of mitophagic-related genes.

Some reports have described that FOXO1 controls the ubiquitin-proteasome pathway by upregulating FBXO32 and TRIM54.^44^ It is known that PGC-1*α* is able to inactivate FOXO1 by promoting AKT1 expression.^45^ The maintenance of basal mitophagy flux by PGC-1*α* can most be explained by its capacity of buffering ROS-mediated activation of the redox-sensitive autophagy inducer FOXO1. This assumption is reinforced by the evidence that Trolox treatment significantly blocks the FOXO1-mediated expression of mitophagic genes. However, we cannot exclude that the inhibition of ATK expression could be operative upon PPARC1A downregulation, thus contributing to FOXO1 nuclear migration. This aspect is currently under investigation in our laboratory.

Many reports have demonstrated that PGC-1*α* is a key integrator of neuromuscular activity in skeletal muscle.^46, 47^ Our results are in agreement with these data and further corroborate the importance of PGC-1*α* in maintaining skeletal muscle metabolism, likely by assuring the increase of intact mitochondrial mass through the correct balance between biogenesis and mitophagy. In fact, PGC-1*α* deficient cells are not able to complete differentiation, as verified by defective MYOG expression, and are committed to a degenerative process, as demonstrated by increased FBXO32 mRNA.^20, 46^ The importance of basal mitophagy and PGC-1*α* in muscle protection was also verified with siPGC-1*α*/siPINK1 co-transfection experiments. In particular, PGC-1*α*/PINK1-deficient cells showed a very high inability to differentiate and increased muscle degeneration. Also, the sole inhibition of mitophagy represents a detrimental process to skeletal muscle cells, which undergo muscle atrophy, although to a lesser extent than PGC-1*α*/PINK1-deficient cells. These data suggest that the inhibition of PGC-1*α* and/or mitophagy induces an accumulation of dysfunctional organelles that, in the end, would lead to skeletal muscle atrophy. Moreover, the data obtained after PINK1 downregulation indicate that basal mitophagy is essential for appropriate differentiation program. Work is in progress in our laboratory, in order to deeply dissect the role of PINK1 in myogenesis and skeletal atrophy.

**Figure 8:**
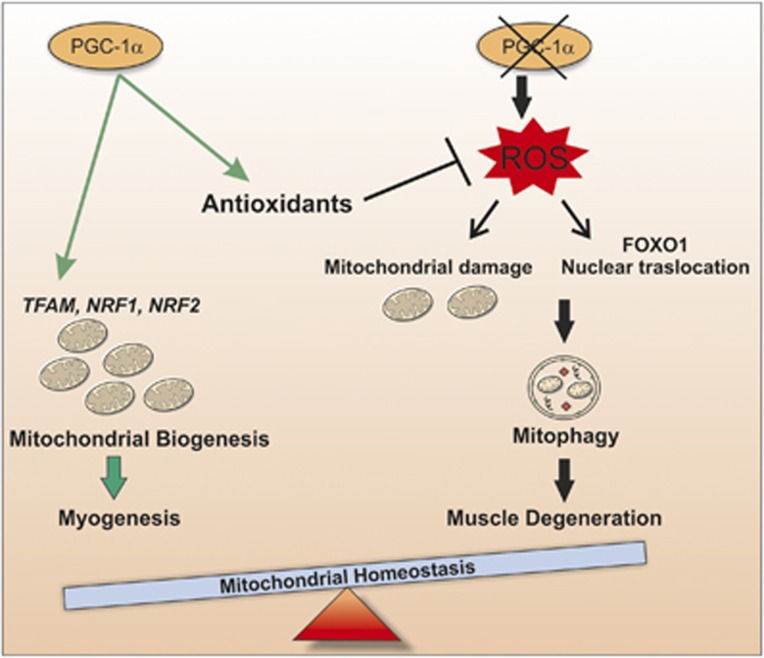
Schematic model of PGC-1*α* function in regulation of mitochondrial content during myogenesis. During myogenesis, PGC-1*α* maintains mitochondrial homeostasis ensuring mitochondrial biogenesis, counteracting oxidative stress and buffering mitophagy. Through this regulation, PGC-1*α* functions as a positive regulator of mitochondrial biogenesis moving the skeletal muscle balance towards the production of new mitochondria. Conversely, the downregulation of PGC-1*α* leads to alteration of mitochondrial network promoting the induction of mitophagy through a ROS-dependent binding of FOXO1 transcription factor on LC3 and PINK1 gene promoter

In this work, we give proof of the involvement of PGC-1*α* in maintaining the correct mitochondrial content during differentiation by buffering ROS production, which can damage mitochondria network causing their degradation *via* mitophagy. At least during myogenesis, PGC-1*α* triggers the increase of mitochondrial mass on one hand by promoting mitochondrial biogenesis and on the other hand by buffering the ROS/FOXO1-mediated expression of genes involved in mitophagy (Figure 8). Thus, the development of therapeutic strategies, which increase muscle PGC-1*α* expression, may be beneficial for maintenance and/or regeneration of skeletal muscle mass in patients with various muscle-wasting conditions.

## Materials and Methods

### Reagents

Protease inhibitor cocktail (P8340), DNase I (D5025), anti-TUBB (T5201), anti-LC3I-II (L7543), Triton X-100 (93443), 6-hydroxy-2,5,7,8-tetramethylchroman-2-carboxylic acid (Trolox) (391913), qPCR primers, TRI Reagent (T9424), IGEPAL CA-630 (I8896), Nonidet P-40 (74385), protein G-Agarose (P7700), Percoll (P1644), chloroquine (CQ, C6628), 3MA (M9281), JC-1 (5,5′,6,6′-tetrachloro-1,1′,3,3′-tetraethylbenzimidazolocarbocyanine iodide) (T4069) polyclonal anti-H2B (SAB4501372) and paraformaldehyde (P6148) were from Sigma-Aldrich (St. Louis, MO, USA). UltraLink streptavidin beads (20347) were from Thermo Fisher Scientific Inc (Rockford, IL, USA). Rabbit polyclonal anti-SOD2 (AB10346) was from Merck Millipore (Darmstadt, Germany). Monoclonal and polyclonal anti-PGC-1*α* (sc-13067), anti-SQSTM1 (sc-25575), anti-PINK1 (sc-33796), anti-PARK2 (sc-30130), anti-TOMM20 (sc-11415), anti-BNIP3 (sc-56167), anti-FOXO1 (sc-374427), anti-COX4I1 (sc-69359), anti-MFN2 (sc-50331), anti-Sp1 (sc-59), anti-LDH (sc-33781), anti-p-RPS6KB1 (sc-7984-R), anti-p-PRKAA2 (sc-101630), anti-PRKAA2 (sc-25792), anti-HSPA9 (sc-13967), anti-TRX1 (sc-18215) and goat monoclonal anti-TFAM (sc-23588) were from Santa Cruz Biotechnology (Santa Cruz, CA, USA). Goat anti-mouse (172–1011) and anti-rabbit (172–1019) IgG (H+L)-horseradish peroxidase conjugated were from Bio-Rad Laboratories (Hercules, CA, USA). 2′,7′-Dihydrodichlorofluorescin diacetate (H_2_DCF-DA, D399), Alexa Fluor 594 Goat Anti-Rabbit IgG (H+L) (A-11012), Hoechst 33342 (H1399) and MitoTrackerRed (M-7512) were from Life Technologies Ltd (Paisley, UK). M-MLV (M1701) and WizardSV Genomic DNA purification kit (A2360) were purchased from Promega (Madison, WI, USA). Ex TAq qPCR Premix (RR420A) was purchased from Lonza (Lonza Sales, Basel, Switzerland). All other chemicals were obtained from Merck Ltd (Darmstadt, Germany).

### Cell culture and treatments

The murine skeletal muscle cell line C2C12 was obtained from the European Collection of Cell Cultures (91031101) (Salisbury, United Kingdom). These cells are able to undergo differentiation into spontaneously contracting myotubes after growth factor withdrawal. Myoblasts were cultured in growth medium composed of Dulbecco's modified eagle's medium (Lonza, BE12-614F) supplemented with 10% fetal bovine serum (Lonza, DE14-802F), 2 mM L-Glutamine (Lonza, 17-605E) and maintained at 37 °C in an atmosphere of 5% CO_2_ in air. Cells were plated at 80% of confluence and cultured in growth medium for 24 h. To induce differentiation, cells were washed in PBS and growth medium was replaced with differentiation medium (DM), which contained 2% heat-inactivated horse serum (Lonza, ECS0090D).

Trolox (200 *μ*M) was added to cells at day 0 and re-added fresh at every medium change, until day 6, when differentiation program was complete. Chloroquine (CQ 5 *μ*M) was added to cells at day 0 for 48 h (day 2). 3-MA (5 mM) was added to cells at day 0 for 48 h (day 2).

### Transfection

C2C12 cells were transfected with a siRNA duplex directed against the following mouse PGC-1*α* (SASI_Mm01_00082035), FOXO1 (SASI_Mm01_00046446) or PIKN1 (SASI_Mm01_00041067) target sequences ((PGC-1*α*(−), FOXO1(−) or PINK1(−) cells). Transfection with a scramble siRNA duplex (scr), with no homology to other mouse mRNA, was used as control. C2C12 cells were also transfected with EGFP-LC3 containing plasmid for co-localization experiments. EGFP-LC3 plasmid was kindly provided by Prof. Francesco Cecconi, Department of Biology, University of Rome ‘Tor Vergata', Italy. Finally, C2C12 cells were transiently transfected with the Addgene plasmid pSV-PGC-1*α* (PGC-1*α*(+) cells) (Addgene, Cambridge, MA, USA) or with empty vector (Mock cells). Transfection efficiency was estimated by co-transfecting the cells with pMAX-FP-Green C vector (Lonza Sales). Cells were transfected by electroporation as described previously^48^ and transfection efficiency of siRNA was evaluated by co-transfecting siRNAs with nonspecific rhodamine-conjugated oligonucleotides. Only experiments that gave transfection efficiency of 80% were considered. Twenty-four hours after transfection (day 0), differentiation was induced.

### Isolation of mitochondria

Crude mitochondria from C2C12 cells were obtained as previously described.^6^ Briefly, the enriched fraction of mitochondria was purified on Percoll gradient according to the protocol from Pellon-Maison *et al.*^49^ To eliminate the possible presence of nuclear DNA contaminants, Percoll-purified mitochondria were incubated for 1 h at 4 °C in the presence of DNase I (20 units/mg) and reaction was stopped by addition of 5 mM EDTA, pH 8.0.

### Isolation of nuclei

Cell pellets were resuspended in lysis buffer containing 10 mM NaCl, 3 mM MgCl_2_, 10 mM Tris-HCl, pH 7.8, 0.5% NP-40, 1 mM DTT and protease inhibitors. Nuclei were collected by centrifugation at 2000 × *g* for 5 min at 4 °C, resuspended in 50 *μ*l of HSB buffer (50 mM Tris-HCl, pH 7.5, 400 mM NaCl, 1 mM EDTA, 1 mM Triton X-100, 0.5% NP-40, 10% glycerol and protease inhibitors) and incubated 30 min on a rotating wheel at 4 °C. The supernatant fraction represents the cytoplasmatic fraction. Pellets were then centrifuged at 22 000 × *g* to remove nuclear debris and the supernatants (nuclear proteins) were used for western blot analysis.

### Western blot analysis

Isolated mitochondria or cell pellets were resuspended in RIPA buffer (50 mM Tris-HCl, pH 8.0, 150 mM NaCl, 12 mM deoxycholic acid, 0.5% Nonidet P-40 and protease inhibitors) or in lysis buffer (10 mM Tris-HCl, pH 7.4, 5 mM EDTA, 50 mM NaCl, 0.5% IGEPAL CA-630 and protease inhibitors), respectively. Protein samples were used for SDS-PAGE followed by western blotting. Nitrocellulose membranes were stained with primary antibodies against COX4I1 (1 : 500), MFN2 (1.500), TUBB (1 : 1000), PGC-1*α* (1 : 500), TRX1 (1 : 1000), TOMM20 (1 : 1000), TFAM (1 : 1000), SOD2 (1 : 2000), FOXO1 (1 : 1000), p-RPS6KB1 (1 : 1000), p-PRKAA2 (1 : 1000), PRKAA2 (1.1000), LDH (1 : 5000), PINK1 (1 : 1000), PARK2 (1 : 1000), BNIP3 (1 : 1000), LC3I-II (1 : 000), SQSTM1 (1 : 2000) and Sp1 (1 : 500). Afterward, the membranes were incubated with the appropriate horseradish peroxidase-conjugated secondary antibody, and immunoreactive bands were detected by a Fluorchem Imaging System upon staining with ECL Select Western Blotting Detection Reagent (GE Healthcare, Pittsburgh, PA, USA; RPN2235). Immunoblots reported in the figures are representative of at least four experiments that gave similar results. TUBB or TOMM20 were used as the loading control. The possible presence of protein contaminants after isolation of mitochondria was measured by incubating nitrocellulose membrane with anti-H2B.

### Determination of protein carbonylation

Carbonylated proteins were detected using the OxyBlot Kit (Millipore, S7150) as previously described.^50^ Briefly, 10 *μ*g of total proteins were reacted with 2,4 dinitrophenylhydrazine (DNP) for 15 min at 25 °C. Samples were resolved on 10% SDS-polyacrylamide gels and DNP-derivatized proteins were identified by western blot analysis using an anti-DNP antibody and an appropriate horseradish peroxidase-conjugated secondary antibody.

### RT-qPCR analysis

Total RNA was extracted using TRI Reagent. Three micrograms of RNA was used for retrotranscription with M-MLV (Promega). qPCR was performed in triplicates by using validated qPCR primers (BLAST), Ex TAq qPCR Premix and the Real-Time PCR LightCycler II (Roche Diagnostics, Indianapolis, IN, USA). mRNA levels were normalized to RPL mRNA, and the relative mRNA levels were determined by using the 2^−ΔΔCt^ method. The primer sequences are listed in Table 1.

### Determination of mitochondrial mass

DNA was isolated by Wizard SV Genomic DNA purification kit (Promega). qPCR was performed in triplicates by using validated qPCR primers (BLAST), Ex TAq qPCR Premix (Lonza, Sales) and the Roche Real-Time PCR LightCycler II (Roche Applied Science, Mannheim, Germany). D-Loop levels were normalized to genomic D-Loop value was normalized to RPL and the relative levels were determined by using the 2^−ΔΔCt^ method. The primers sequences are listed in Table 1. Alternatively, mitochondrial mass was detected by cytofluorimetric analysis after incubation of cells with 100 nM MitoTracker Red for 30 min, as previously reported.^51^

### mtDNA immunoprecipitation assay (mtDNA-IP)

mtDNA immunoprecipitation was performed as described previously^6^ by using qPCR analysis to quantify the promoter binding with 30 cycles total (95 °C, 1 s; 60 °C, 30 s; 72 °C, 60 s). Results are expressed as percentage of Input (1%) values. The primers used are reported in Table 1.

### Evaluation of ROS content

ROS were detected by cytofluorimetric analysis after incubation for 30 min at 37 °C with 50 *μ*M H_2_DCF-DA as previously reported.^52^ The fluorescence intensity of 10 000 cells from each samples were analyzed by FACScalibur instrument (Beckton-Dickinson, San Jose, CA, USA). Data were analyzed using the WinMDI 2.8 software (Scripps Research Institute, La Jolla, CA, USA).

### Microscopy analysis

Cells were seeded directly on glass coverslips and after 48 h were fixed with 4% paraformaldehyde and permeabilized by incubation with 0.2% Triton X-100. For mitophagy analysis, C2C12 cells were co-transfected with scramble (scr) or PGC-1*α* siRNA (PGC-1*α*(−)) and EGFP-LC3 and incubated with mouse HSPA9 (1 : 50) antibody. After staining with the appropriate AlexaFluor-conjugated secondary antibodies (1 : 1000), cells were incubated with Hoechst 33342 (Life Technologies, Carlsbad, CA, USA) to visualize nuclei. Images were visualized with an Olympus Fluoview 1000 confocal laser scanning system (Segrate, Milano, Italy). The images presented were captured under constant exposure time, gain and offset. Pearson's correlation coefficient, *R*(*r*), was calculated for fluorescence intensities of both the confocal channels. This coefficient describes the correlation between the intensity distribution or pattern overlap in two channels in terms of a least squares fit. This value can be between −1 and 1, and *R*=1 indicates complete correlation between the two channels. Finally, the overlap coefficient indicates an overlap of the signals and thus represents the true degree of colocalization.

### Assessment of mitochondrial membrane potential (ΔΨm) using the fluorescent indicator JC-1

C2C12 myotubes were grown on glass coverslips and incubated with 1 *μ*g/ml JC-1 for 30 min at 37 °C. Cells were subsequently fixed with paraformaldehyde for 10 min and then stained with Hoechst 33342 to visualize nuclei. Finally, cells were washed three times with PBS and analyzed by fluorescence microscopy. The images presented were captured under constant exposure time, gain and offset.

### Statistical analysis

The results are presented as means±S.D. Statistical evaluation was conducted by ANOVA, followed by the post-Student-Newman-Keuls test. Differences were considered to be significant at *P*<0.05.

## Figures and Tables

**Table 1:** List of primers used for RT-qPCR and ChIP analysis

**Gene**	**Sequences**
COX4I1 (mouse)	RV 5′-TCACAACACTCCCATGTGCT-3′ FW 5′-GAATGTTGGCTTCCAGAGCG-3′
PGC-1*α* (mouse)	RV 5′-ACTCGGATTGCTCCGGCCCT-3′ FW 5′-ACTGACGGCCTAACTCCACCCA-3′
MT-CO1 (mouse)	RV 5′-ACCTATTCGTACTCCGGCCT-3′ FW 5′-ATGTGACCCGATTTGGCGTT-3′
MT-ATP6 (mouse)	RV 5′-GTGGAAGGAAGTGGGCAAGTGAGC-3′ FW 5′-GCCATTCCACTATGAGCTGGAGCC-3′
TFAM (mouse)	RV 5′-TCCGCCCTATAAGCATCTTG-3′ FW 5′-CCGAGGTGGTTTTCATCTGT-3′
PARK2 (mouse)	RV 5′-ACAGGGCTCCTGACATCTG-3′ FW 5′-CAAGGACACGTCGGTAGCTT-3′
LC3 (mouse)	RV 5′-CCACTCTTTGTTCAAAGCTCCGGC-3′ FW 5′-CGTCGCCGGAGTCAGATGTC-3′
NRF1 (mouse)	RV 5′-ATGGGCGGCAGCTTGACTGT-3′ FW 5′-GCGCAGCCGCTCTGAGAACTTAT-3′
NRF2 (mouse)	RV 5′-TGGGCCCTGATGAGGGGCAGTG-3′ FW 5′-TCCGCCAGCTACTCCAGGTTGG-3′
POLRMT (mouse)	RV 5′-GACGGCGTTAGGTTGACTGA-3′ FW 5′-CCAGTTCACCAGGATGGCTC-3′
POLG (mouse)	RV 5′-ACAAGTCCTCGGTCCGCTTC-3′ FW 5′-CACCCTGAGGCTGCGTG-3′
RPL (mouse)	RV 5′-ATGGCGGAGGGGCAGGTTCTG-3′ FW 5′-GTACGACCACCACCTTCCGGC-3′
BNIP3 (mouse)	RV 5′-GGTCGACTTGACCAATCCCAT-3′ FW 5′-ACAGCACTCTGTCTGAGGAA-3′
D-Loop *consensus sequence*	RV 5′-CATGAATAATTAGCCTTAGGTGAT-3′ FW 5′-TCAGACATCTGGTTCTTCTTACTTCAG-3′
FOXO1 *consensus sequence* on LC3 promoter	RV 5′-CCCAAGGATCTCAACCAAAC-3′ FW 5′-CCTCAGCTGGTAAGAGCAT-3′
FOXO1 *consensus sequence* on PINK1 promoter	RV 5′-CTGTCGACCGCCATGGTGGCGCGGTGACC-3′ FW 5′-TGAGAGCACTTGGGAGTGGGGGAGAAGAG-3′

## Abbreviations

AKT1: v-akt murine thymoma viral oncogene homolog 1
ATG4: autophagy-related 4B, cysteine peptidase
BNIP3: BCL2/adenovirus E1B 19 kDa interacting protein 3
CAT: catalase
Cox4I1 (COX4): cytochrome *c* oxidase subunit IV isoform 1
DNC: decorin
FBXO32 (FBXO32): F-box protein 32
FOXO1, 3: forkhead box O1, O3
GPX1: glutathione peroxidase 1
HSPA9: heat shock 70 kDa protein 9 (mortalin)
LC3I-II: microtubule-associated protein 1 light chain 3 alpha
MFN2: mitofusin 2
MYOG: myogenin (myogenic factor 4)
MT-ATP6: mitochondrially encoded ATP synthase 6
MT-CO1: mitochondrially encoded cytochrome *c* oxidase I
mtDNA: mitochondrial DNA
MTOR: mechanistic target of rapamycin (serine/threonine kinase)
NFE2L2: nuclear factor, erythroid 2-like 2
NRF1,2: nuclear respiratory factor 1 and 2
PARK2 (Parkin): parkin RBR E3 ubiquitin protein ligase
PGC-1*α*: peroxisome proliferator-activated receptor gamma coactivator 1 *α*
PINK1: PTEN induced putative kinase 1
POLG: polymerase (DNA), gamma
PRKAA2 (AMPK): protein kinase, AMP-activated, alpha 2 catalytic subunit
POLRMT: polymerase (RNA) mitochondrial (DNA directed)
RPL: ribosomal protein large subunit
RPS6KB1: ribosomal protein S6 kinase, 70 kDa, polypeptide 1
SOD2: superoxide dismutase, mitochondrial
SQSTM1: sequestosome 1
TFAM: transcription factor A, mitochondrial
TRIM54 (MURF1): tripartite motif containing 54
TRX1: thioredoxin
TUBB: tubulin, beta class I
