# Supplementary figures and images for: PGC-1α buffers ROS-mediated removal of mitochondria during myogenesis
Source: Cell Death Dis. 2014 Nov 6;5(11):e1515–. doi: 10.1038/cddis.2014.458 (PMC4260723; doi:10.1038/cddis.2014.458)

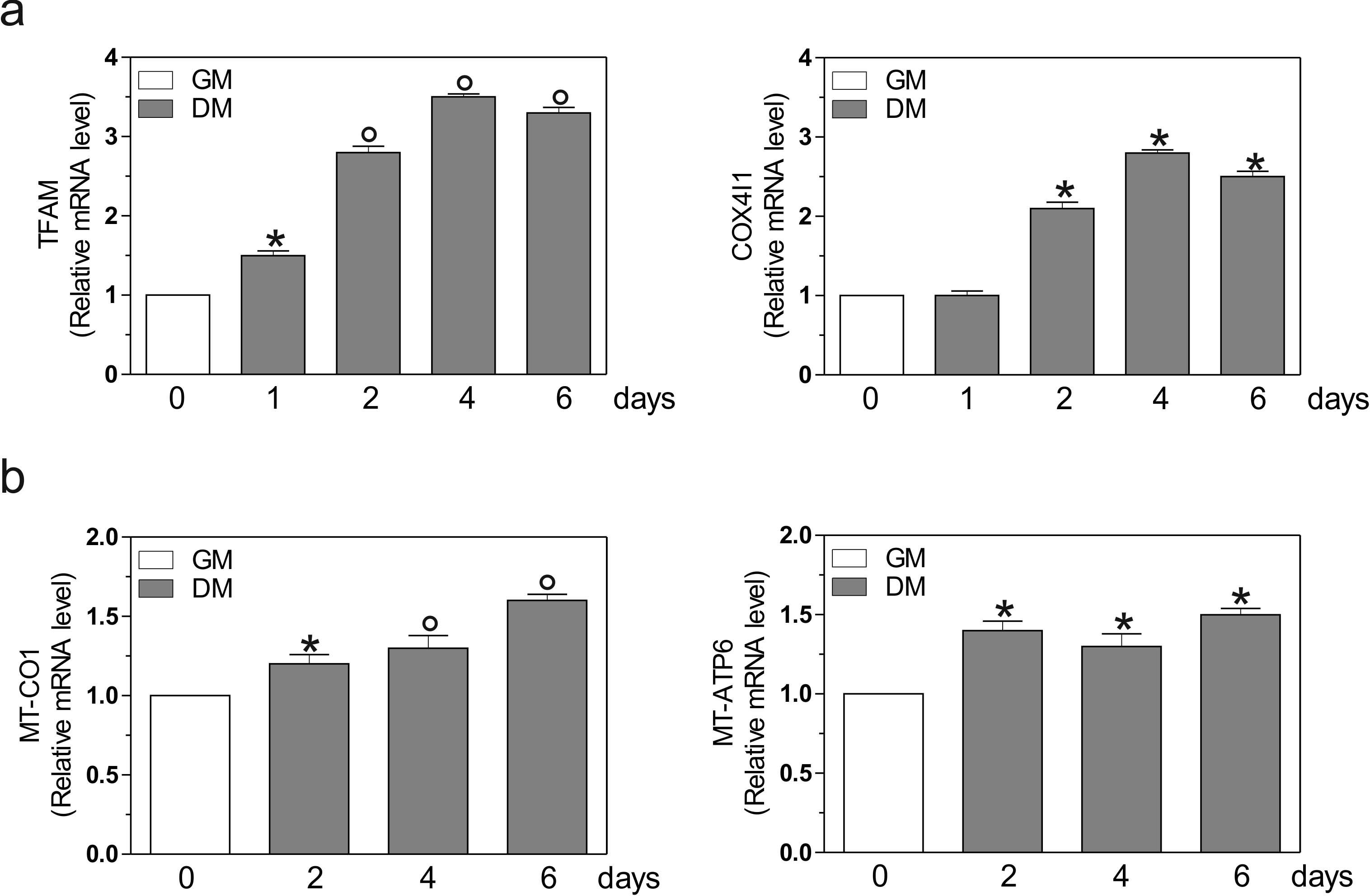

Supplement: Supplementary Figure 1 [file cddis2014458x1.tif]

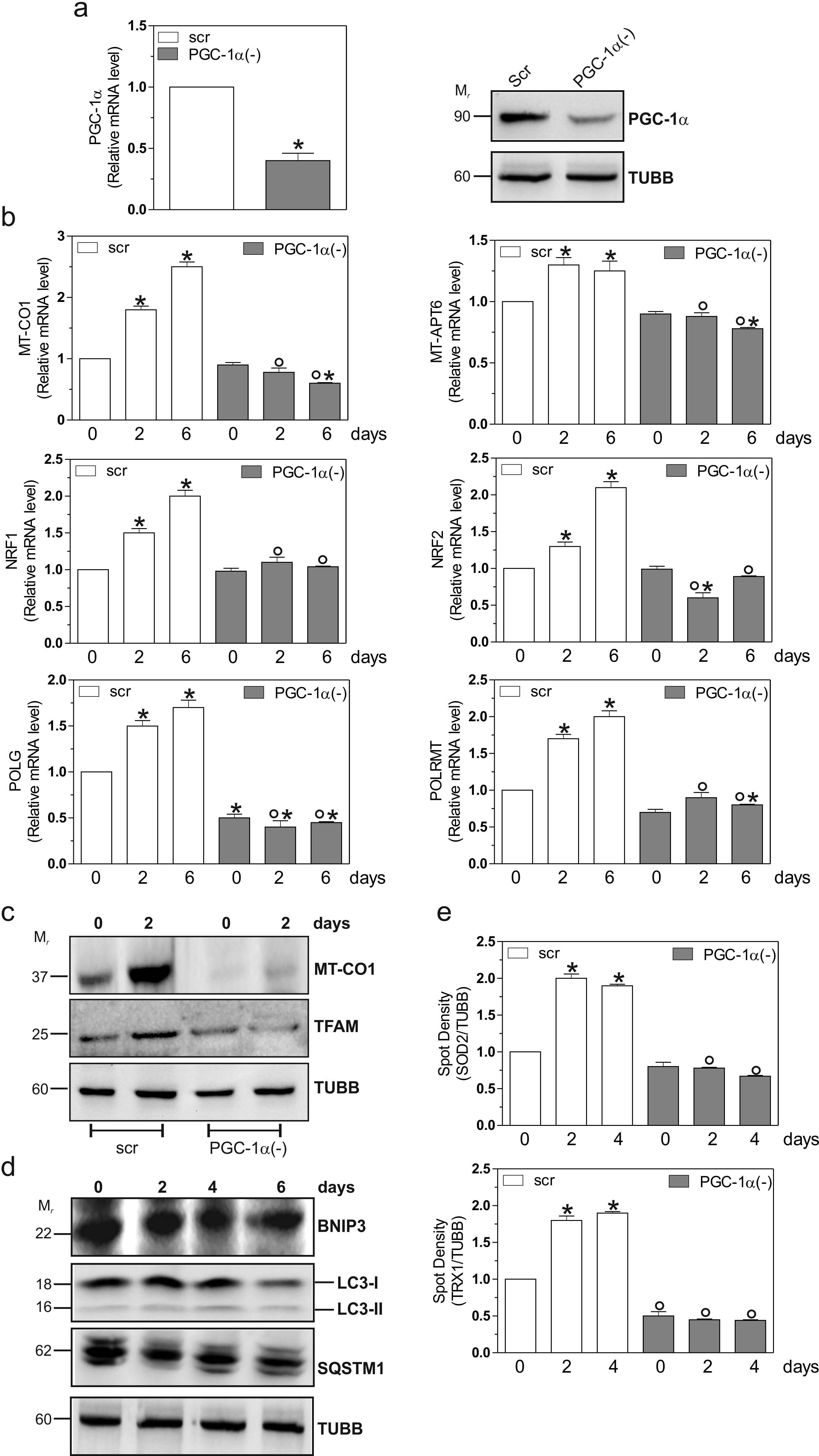

Supplement: Supplementary Figure 2 [file cddis2014458x2.tif]
