# Supplementary material for: PGC-1α buffers ROS-mediated removal of mitochondria during myogenesis
Source: Cell Death Dis. 2014 Nov 6;5(11):e1515–. doi: 10.1038/cddis.2014.458 (PMC4260723; doi:10.1038/cddis.2014.458)
Supplement: Supplementary Figures legend [file cddis2014458x3.docx]

*Supplementary Figure 1.* **Myogenesis promotes an induction of PPARGC1A co-transcriptional activity.**

C2C12 cells were differentiated in DM for the indicated days. (a) Total RNA was isolated and relative mRNA levels of TFAM and COX4I1 were analyzed by RT-qPCR. Data are expressed as means ± S.D. (n=6, *p<0.01, °p<0.001 vs day 0). (b) Total RNA was isolated and relative mRNA levels of MT-CO1 and MT-ATP6 were analyzed by RT-qPCR. Data are expressed as means ± S.D. (n=6, *p<0.01, °p<0.001 vs day 0).

*Supplementary Figure 2*. **PPARGC1A deficiency induces a decrease of mitochondrial biogenesis and antioxidant enzymes.**

Cells were transfected with scramble (scr) or PPARGC1A siRNA [PPARGC1A(-)] and differentiated in DM for the indicated days. (a) Total RNA was isolated, and relative mRNA levels of PPARGC1A were analyzed by RT-qPCR. Data are expressed as means ± S.D. (n=5, *p<0.001) (left panel). Twenty µg of total proteins were loaded for Western blot analysis of PPARGC1A (right panel). TUBB was used as loading control. (b) Total RNA was isolated, and relative mRNA levels of MT-CO1, MT-ATP6, NRF1, NRF2, POLG and POLRMT were analyzed by RT-qPCR. Data are expressed as means ± S.D. (n=4, *p<0.001 vs day 0 scr cells; °p<0.001 vs scr cells). (c) Twenty µg of total proteins were loaded for Western blot analysis of MT-CO1 and TFAM. TUBB was used as loading control. (d) Twenty µg of total proteins were loaded for Western blot analysis of BNIP3, LC3I-II and SQSTM1. TUBB was used as loading control. (e) Density of immunoreactive bands was calculated using the software Quantity one (Bio-Rad) and data are shown as ratio of SOD2/TUBB and TRX1/TUBB. Data are expressed as means ± S.D. (n=4; *p<0.001 vs day 0 scr cells; °p<0.001 vs scr cells). Immunoblots reported in the figures are representative of at least four experiments that gave similar results.
